# Effects of Water on Structure and Dynamics of Trehalose Glasses at Low Water Contents and its Relationship to Preservation Outcomes

**DOI:** 10.1038/srep28795

**Published:** 2016-07-08

**Authors:** Lindong Weng, Shima Ziaei, Gloria D. Elliott

**Affiliations:** 1 Department of Mechanical Engineering and Engineering Science, University of North Carolina at Charlotte, Charlotte, NC 28223, USA.

## Abstract

Dry preservation of biologics in sugar glasses is regarded as a promising alternative to conventional cryopreservation. Evidence from various studies has suggested that there is a critical range of water content beyond which the viability of preserved biologics can be greatly compromised. In this study the viability of T-cells was determined as a function of end water content after microwave-assisted drying in trehalose solutions. Hydrogen-bonding and clustering phenomena in trehalose solutions of the same moisture content were also evaluated using molecular dynamics simulation. Post-rehydration viability decreased dramatically within the range of 0.1–1 gH_2_O/gdw. Molecular modeling revealed that as the water content approached 0.1 gH_2_O/gdw the matrix formed a large interconnected trehalose skeleton with a minimal number of bound water molecules scattered in the bulk. The diffusion coefficients of trehalose oxygen atoms most distant from the glycosidic linkage fluctuated around 7.5 × 10^−14^ m^2^/s within the range of 0.02–0.1 gH_2_O/gdw and increased again to ~1.13 × 10^−13^ m^2^/s at 0.01 gH_2_O/gdw and below due to the loss of water in the free volume between trehalose molecules. These insights can guide the optimal selection of final moisture contents to advance dry preservation methods.

Dry preservation enables the long-term storage of biologics at non-refrigerated temperatures and is regarded as a cost-effective alternative to cryopreservation. Because cryogenic temperatures are not required, biological samples preserved in the dried state also offer more storage and transportation flexibility, circumventing the need for shipping on dry ice or in liquid nitrogen shippers^1^. Dry preservation is facilitated by suspending the biological material in sugar-based compositions that can be vitrified by various dehydration methods. Among the lyo-protective sugars, trehalose is the most commonly used due to its high glass transition temperature (*T*_*g*_) and the exceptional capability of hydrogen-bonding with biomacromolecules^2^. It has been proposed that trehalose can substitute for water under low moisture content conditions via interactions with lipid bilayers or proteins, thereby maintaining their native structure^3^,^4^. Considering these advantages, trehalose has been explored as a nontoxic protective additive to preserve various biologics including human adipose-derived stem cells^2^,^5^ and tissue-engineered skin^6^.

Despite the utility of sugar glasses for biopreservation purposes, there seems to be a critical range of water content beyond which the viability of certain preserved biological samples is greatly compromised. When creating vitrified products from sugar solutions, the lower the water content of the vitrified sample the higher the *T*_*g*_ that is achieved. But a drier state of the preserved matrix might not necessarily produce a better preservation outcome^4^,^7^,^8^,^9^,^10^,^11^,^12^. For example, it has been found that when the water content remained above ~0.2 gH_2_O/gdw (grams of water per gram of dry weight), mouse sperm dried with an initial medium of 0.5 M trehalose and EGTA and stored at 4 °C retained a high level of developmental potential towards the blastocyst stage. The protective effect conferred by trehalose was significantly diminished when the water content decreased to an anhydrobiotic level of ≤0.1–0.3 gH_2_O/gdw^12^. A similar phenomenon was also observed recently by Liu *et al*.^10^ when they dried and stored mouse sperm in a matrix containing 3-O-methyl-D-glucose (3-OMG). They proposed that a small amount of water was required to preserve the sperm functionality during desiccation and storage^10^. These and other observations have generated much discussion on the idea of critical water content for biologics that relates to the state of water in the vicinity of biological structures.

It has also been observed that the gel to liquid crystalline phase transition temperature (*T*_*m*_) of phospholipids does not change significantly until the water content falls below ~0.25 gH_2_O/gdw^13^. Below this critical level, the *T*_*m*_ can increase sharply as a result of a denser packing of the lipid headgroups because the water molecules are depleted from the vicinity of the headgroups and the lipid membranes can be destabilized^13^. For example, when dried through this water content, the *T*_*m*_ of DPPC can be elevated from 41 °C to ~110 °C, and that of egg PC can rise from −1 °C to about 70 °C^14^. This change can lead to leakage of solutes across the membrane. Pauling^15^ also suggested that proteins stored in lyophilized form should not be excessively dried. The hydration shell has been suggested to be necessary for conserving certain highly polar residues on the protein surface and thus avoiding denaturation^9^,^15^. Drying methods such as microwave-assisted^16^,^17^, spin^1^, or air^11^ drying, are able to deplete macromolecules and membranes of their hydration shell water^13^,^18^. This allows an extremely low water content to be achieved and, as a result, the glass transition temperature of the sugar-water matrix can be elevated above the temperature of drying. For example, Aksan and Toner^19^ have demonstrated that vitrification of trehalose-water compositions can be achieved by isothermal dehydration. Because of the possibility of depleting biologically essential water or structural water during dehydration, it is important to understand the changes in both the glassy matrix and the state of embedded biomolecules at low moisture contents.

Despite recent progress in understanding the drying characteristics of sugar-based preservation media, there are still some fundamental, unanswered questions. For example, what is the critical water content at which all water molecules are hydrogen-bonded to solutes or other macromolecules? What is the basis for the non-monotonic relationship between molecular mobility and water content? Molecular dynamics studies of aqueous trehalose systems to date have not concentrated on the low water content composition range. Before exploring more complex phenomena such as dehydration-induced denaturation of proteins or damage to membranes in a particular water content regime, it is of fundamental importance to examine the structure and dynamics of the bulky drying media at water contents where many biological products are likely to be stored. In the present study we conducted molecular dynamics simulations to investigate the hydrogen-bonding characteristics in aqueous trehalose mixtures with water contents within the range of 0–2 gH_2_O/gdw. The hydrogen-bonding characteristics amongst trehalose molecules and between trehalose and water molecules were quantitatively studied, including clustering phenomenon. To provide further and direct evidence for the existence of the critical water content range when biological samples are subject to dehydration conditions, we also carried out viability studies on Jurkat Clone E6-1 cells that had been dried in trehalose solutions to various end water contents by employing a microwave-assisted drying approach.

## Results and Discussion

**Figure 1:**
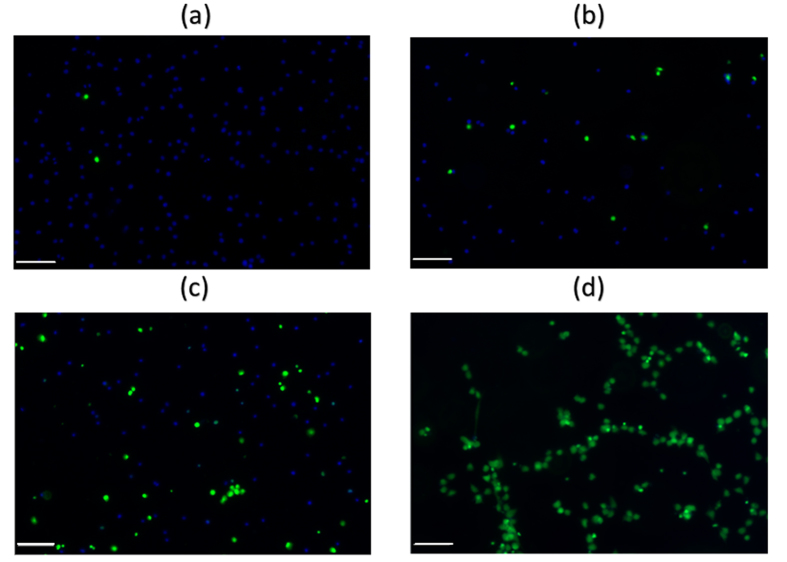
Representative micrographs of (**a**) unprocessed control cells, and cells dried in 300 mM trehalose to (**b**) 1.11 gH_2_O/gdw, (**c**) 0.27 gH_2_O/gdw, and (**d**) 0.01 gH_2_O/gdw. All cells were stained with Hoeschst 33342 (blue) and cells with a compromised membrane were stained with Sytox (green). Micrograph bar represents 50 microns.

Jurkat E6-1 cells were dried in 1) 200 mM trehalose in 0.33X PBS and 2) 300 mM trehalose, using a microwave-assisted drying method. This method is applicable to adherent and non-adherent cells, but as is typical for cryopreservation methods, cells are detached and suspended prior to preservation. Solidification of cells in an adherent state can induce significant mechanical stress to cells during processing and is generally avoided. Microwave-assisted drying is recognized as a fast, uniform and reproducible method to dehydrate biomaterials into the glassy state^20^,^21^, and it has been successfully applied to dry preserve cat germinal vesicles^17^. Representative micrographs showing the distribution of total cells, stained with Hoechst 33342 and membrane compromised cells, stained with Sytox Green, are shown in Fig. 1 for cells dried in 300 mM trehalose to a range of moisture contents. Unprocessed control cells, as shown in Fig. 1(a), are observed to have intact membranes. As drying progresses towards lower moisture contents the number of membrane compromised cells begins to increase as shown for moisture contents of 1.11 gH_2_O/gdw, Fig. 1(b), and 0.27 gH_2_O/gdw, Fig. 1(c). When the moisture loss progresses to 0.01 g H_2_O/gdw it is clear from Fig. 1(d) that the cells are becoming increasingly sequestered into narrow channels, and all membranes are compromised. Cellular staining is also more diffuse, consistent with compromised cellular architecture. Observations were similar for cells dried in both solutions.

**Figure 2:**
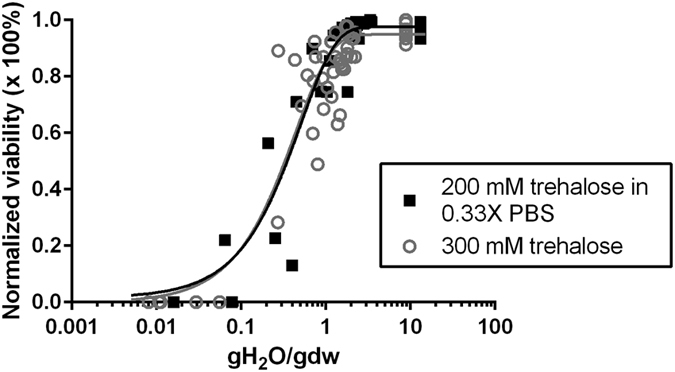
The normalized viability of Jurkat T cells dried to various end moisture contents. The data points were fitted by the Gaussian equation, 
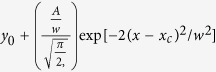
 with *y*_0_, *A, w*, and *x*_c_ as fitting parameters. *R*^2^ = 0.8508 for 200 mM trehalose plus 0.33X PBS and *R*^2^ = 0.8036 for 300 mM trehalose.

The post-rehydration viability as a function of the end water content was quantified by determining the number of membrane intact cells, compared to the total number of cells, and the data plotted as shown in Fig. 2. In both compositions the post-rehydration viability decreased dramatically as the moisture content decreased from 2 to 0.1 gH_2_O/gdw. The Effective Concentration of water that results in 50% cells death (EC_50_) was determined to be 0.35 gH_2_O/gdw for the 200 mM trehalose plus 0.33X PBS solution, and 0.33 gH_2_O/gdw for the 300 mM trehalose solution, statistically equivalent results. These results demonstrate that the cell membrane integrity was significantly compromised as cells were dried through the 0.1–2 gH_2_O/gdw region. In other studies it has been observed that below ~0.25 gH_2_O/gdw the phase transition temperature of phospholipids can increase dramatically, which results from the increased packing of lipid headgroups due to the removal of hydration water^13^. Trehalose in the drying solutions can substitute for water by hydrogen-bonding with lipid headgroups to maintain the spacing between them and slow down the increase of *T*_*m*_^3^,^4^. But the protection conferred by trehalose is far from sufficient for the cells to survive the drying process. Consequently, as the *T*_*m*_ increases above the drying temperature, the phospholipids can transition from the initial liquid crystalline phase into the gel. Upon rehydration, the *T*_*m*_ decreases below the drying temperature. This gel-to-liquid crystalline phase transition can result in compromise of the integrity of the phospholipid bilayers and adverse solute leakage. Since phospholipids are a major component of all cell membranes, the significant decrease in the membrane integrity as the water content decreases from 2 to 0.1 gH_2_O/gdw can be caused by the removal of hydration water in membranes when cells were dried excessively, but it is also possible that mechanical forces can play a role.

**Figure 3:**
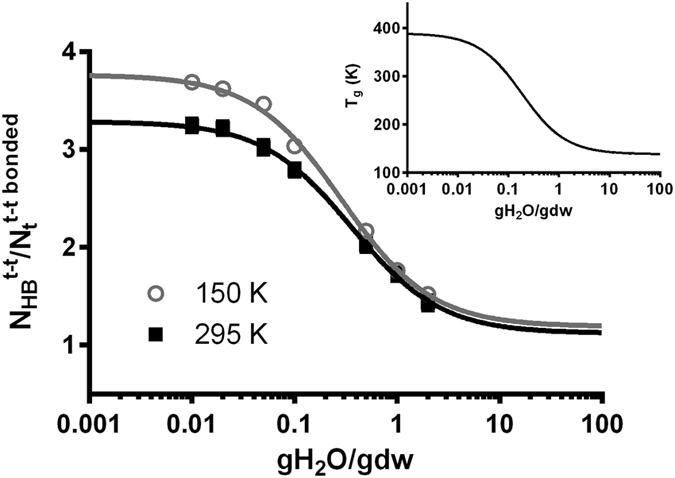
The normalized number (
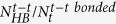
) of intermolecular H-bonds among trehalose molecules as a function of water content (gH2O/gdw) at 150 K and 295 K. The fitting curves are described by Eq. (2). Inset: The glass transition temperature of aqueous trehalose solutions as a function of water content reported in ref. 19, and fit with Eq. (1).

In light of the direct evidence of a critical water content for cell survival, we conducted molecular modeling studies of aqueous trehalose solutions covering the critical range observed in these experiments (0–2 gH_2_O/gdw). Understanding the structural and dynamic characteristics in the bulky drying media is the first step towards investigations on more complicated systems involving proteins or lipid bilayers. In this study, the molecular structure of the trehalose-water matrix was analyzed based on the hydrogen-bonding characteristics. The number of intermolecular H-bonds among trehalose molecules (t-t), denoted as 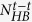, was normalized by the number of trehalose molecules participating in these t-t H-bonds (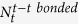). This ratio (
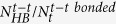
) was plotted as a function of water content, and is shown in Fig. 3. It is evident that at both temperatures investigated the average number of t-t H-bonds per trehalose molecule increases dramatically from ~1 H-bond per trehalose molecule in dilute solution to >3 H-bonds when the water content decreases to 0.1 gH_2_O/gdw. Below 0.1 gH_2_O/gdw, the number of H-bonds begins to level out towards that of completely dehydrated trehalose (i.e. 3.77 ± 0.08 at 150 K and 3.29 ± 0.08 at 295 K). It is interesting that the relationship of 
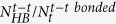
 as a function of water content is similar to the *T*_*g*_ profile of aqueous trehalose mixtures when *T*_*g*_ data is plotted with the *x* axis in log10 scale, as shown in the inset of Fig. 3. The *T*_*g*_ profile of aqueous trehalose solutions is often described by the Gordon-Taylor equation (Eq. (1)).


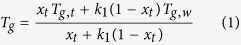


wherein *T*_*g,t*_ and *T*_*g,w*_ are the glass transition temperatures of pure trehalose and pure water, respectively, *x*_*t*_ is the mass fraction of trehalose, and *k*_1_ is the fitting parameter that accounts for the unequal contribution of individual components to the *T*_*g*_. Because of the mathematical similarity in data sets, we obtained the best-fits for the 
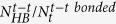
 as a function of moisture content data using a form similar to the G-T equation as shown by Eq. (2).


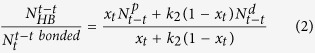


wherein 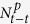 is the average number of t-t H-bonds per bonded trehalose molecule in pure trehalose and 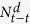 is the average number of t-t H-bonds per bonded trehalose molecule when the solution is infinitely dilute. For pure trehalose the value of 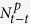 was determined in this study to be 3.77 and 3.29 at 150 K and 295 K, respectively. Both 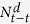 and *k*_2_ were implemented as fitting parameters, and these best-fit values were determined and listed in Table 1. The value of 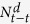 was found to be slightly larger than 1, indicating that when the solution is infinitely dilute, if a trehalose molecule participates in hydrogen bonds with another trehalose molecule (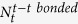 = 2), then it will form, at least, two H-bonds with the other molecule (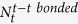 ≥ 2). In other words, 
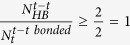
. The similarity in data sets as seen in Fig. 3 implies a close correlation between the extent of the H-bonded trehalose network and the observed glass transition temperature.

With regards to the temperature effect seen in Fig. 3, as expected, the number of trehalose-trehalose H-bonds that each bonded trehalose molecule forms at 150 K will be larger than that at 295 K. At lower temperatures, especially at 150 K, in which all of the compositions under study would be in the glassy state according to the *T*_*g*_ information, the molecular mobility decreases substantially and the volume of the whole matrix is compressed compared to simulations at the equivalent water content but at higher temperatures, leading to more intermolecular H-bonds amongst trehalose molecules compared to those at 295 K.

In addition, it was observed that approximately 97% of the total trehalose molecules formed H-bonds with other trehalose molecules below the water content of 0.1 gH_2_O/gdw. This percentage was only 52.1% at 2.0 gH_2_O/gdw and 150 K and 46.3% at 2.0 gH_2_O/gdw and 295 K (data not shown). In other words, as the dehydration proceeds below 0.1 gH_2_O/gdw, any further depletion of water molecules from the matrix will not alter the bulky structure significantly since the vast majority (≥97%) of trehalose molecules have already joined in a common, interconnected skeleton woven by intermolecular t-t H-bonds. Further removal of water molecules below 0.1 gH_2_O/gdw will only slightly decrease the number of H-bonds between water and trehalose and thus potentially increase the number of H-bonds among trehalose molecules that have already interacted with each other. The formation of the bulky, interconnected trehalose skeleton could be responsible for the slowed-down increase in *T*_*g*_ below 0.1 gH_2_O/gdw and then the eventual leveling out as the matrix approaches complete dehydration.

**Figure 4:**
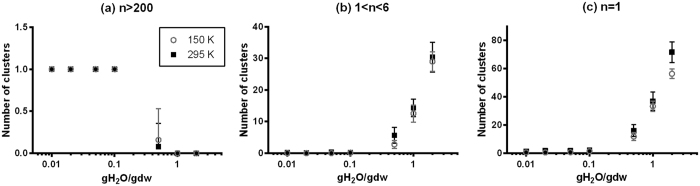
The number of *n*-body trehalose clusters in aqueous trehalose solutions of different water contents. A *n*-body cluster means that there are a total of *n* trehalose molecules in the cluster and each molecule connects with any other ones in the cluster through direct or indirect H-bonds. (a: *n* > 200, b: 1 < *n* < 6, and c: *n* = 1)

The high level of interconnection among trehalose molecules can result in trehalose clusters of various sizes^22^,^23^. Therefore, we calculated the numbers of large and small clusters comprised of only trehalose molecules at different temperatures. It is clear from Fig. 4(a) that below 0.1 gH_2_O/gdw the matrix forms a cluster containing over 200 trehalose molecules (close to the total number of trehalose molecules in the simulation box), indicating that each of these trehalose molecules can connect to any other trehalose molecules in the cluster through direct or indirect t-t H-bonds. However, trehalose molecules in the matrix of >0.1 gH_2_O/gdw tend to form many isolated, small-sized clusters, as seen in Fig. 4(b,c). For example, over 40 single trehalose molecules can be found in the matrix of ≥1 gH_2_O/gdw as seen in Fig. 4(c). These single trehalose molecules are surrounded by water molecules and do not contact any other trehalose molecules.

**Figure 5:**
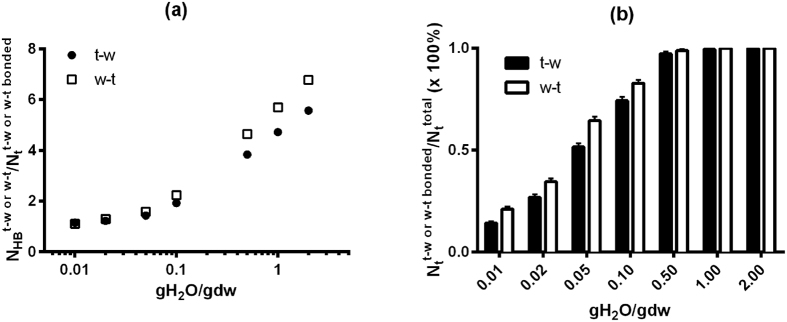
(**a**) The average number of H-bonds between trehalose and water molecules per molecule of H-bonded trehalose, 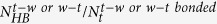, as a function of water content (gH_2_O/gdw) at 150 K. Note that the standard deviation is less than 0.08; (**b**) The percentage of trehalose molecules that are H-bonded to water, 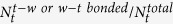, as a function of water content at 150 K.

With increasing water content, the t-t H-bonding capacity will inevitably decrease as seen in Fig. 3 due to more H-bonding interactions between water and trehalose. This is one aspect of what is referred to as the plasticization effect of water. In this study, water molecules that have no contact with trehalose molecules are defined as free water while those interacting with trehalose through H-bonds are referred to as bound water. Specifically, there are two types of H-bonds formed between trehalose and water molecules: 1) H-bonds to which trehalose donates an H atom, denoted as t-w, and 2) H-bonds to which water donates the H atom, denoted as w-t. For either t-w or w-t H-bonds, the stoichiometry, namely the average number of t-w or w-t H-bonds that each participating trehalose molecule forms, descends linearly with decreasing log_10_ (gH_2_O/gdw) until 0.1 gH_2_O/gdw as seen in Fig. 5(a). The ratio of 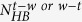 (the number of t-w or w-t H-bonds) and 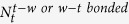 (the number of trehalose molecules that form H-bonds with water) levels off towards 1 below 0.1 gH_2_O/gdw, implying that each bonded trehalose molecule interacts with only one water molecule via a single t-w or w-t H-bond. Moreover, as seen in Fig. 5(b) the trehalose molecules that H-bond with water constitute ~100% of the total trehalose molecules in the simulation box (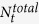) at 0.5 gH_2_O/gdw or above. But below a water content of 0.5 gH_2_O/gdw the proportion begins to decrease gradually as fewer water molecules are available for forming t-w or w-t H-bonds. Or put another way, as the matrix is dehydrating, when the water content is above 0.5 gH_2_O/gdw, the water molecules are able to solvate almost every trehalose molecule in the matrix. The almost 100% participation of trehalose molecules in forming t-w or w-t H-bonds at 0.5 gH_2_O/gdw and above is consistent with the large population of isolated trehalose molecules or small-sized trehalose clusters (*n* < 6) within this range of water content as seen in Fig. 4(b,c). But when the water content goes below 0.5 gH_2_O/gdw, the further removal of water molecules from the matrix will lead to the formation of the abovementioned fully networked trehalose skeleton.

**Figure 6:**
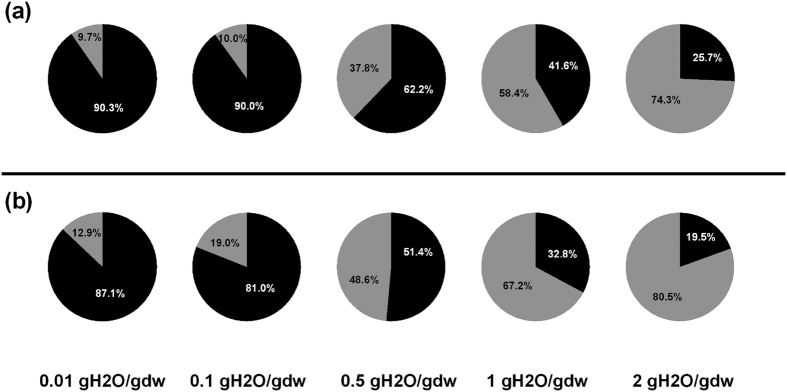
The proportions of bound and free water in trehalose-water matrices at (**a**) 150 K and (**b**) 295 K, respectively. (The standard deviation is less than ±4% in panel (**a**) and is less than ±5% in panel (**b**); black: bound water and grey: free water)

We observe from Fig. 6(a) that the vast majority (≥90%) of water at 150 K is H-bonded to trehalose (i.e. is considered bound water) within the range of 0.01 to 0.1 gH_2_O/gdw and no more than 10% is free in these compositions. When the water content is 0.5 gH_2_O/gdw, however, nearly 40% of the total water molecules have no H-bonding interactions with trehalose molecules, and are considered free water. This proportion of free water increases to almost 75% when the water content is 2 gH_2_O/gdw. The data at 295 K follows the same trend as those at 150 K even though the bound water percentage is slightly lower and the free water percentage is slightly higher at 295 K due to the higher molecular mobility of water molecules at higher temperatures.

**Figure 7:**
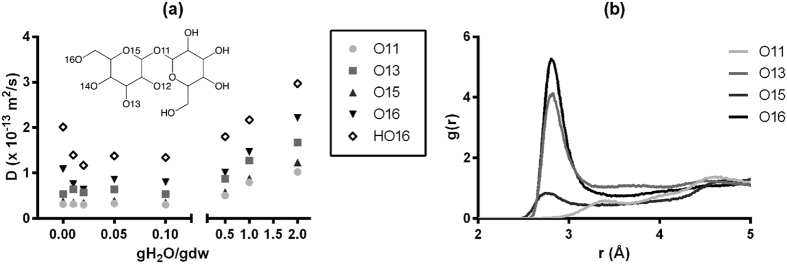
(**a**) The diffusion coefficient (*D*) of different O or H atoms in trehalose as a function of water content at 150 K. Note that the standard deviation is less than 0.07 × 10^−13^; (**b**) The radial distribution functions of O_w_-O_t_ pairs with water content of 0.1 gH_2_O/gdw at 150 K. (O_w_ represents the oxygen atom in water and O_t_ the oxygen atoms in trehalose.)

The change of the bulky structure of aqueous trehalose compositions can affect the molecular mobility of trehalose and thus the stability of the amorphous state, as well as the molecular mobility of structures or molecules contained within this matrix, if any. It is found here that the diffusion coefficient of O or H atoms in trehalose decreases with decreasing water content until 0.1 gH_2_O/gdw, as seen in Fig. 7(a). Given the symmetric structure of the trehalose molecule, in this figure we only present selective O and H atoms in one of the two glucose rings of trehalose. For atoms of O16 and HO16, the diffusion coefficient is found to fluctuate between ~6.2 × 10^−14^ and ~8.2 × 10^−14^ m^2^/s within the range of 0.02–0.1 gH_2_O/gdw and increase again at 0.01 gH_2_O/gdw and below. For atoms of O11, O13 and O15, the diffusion coefficient fluctuates around ~3 × 10^−14^ (O11 and O15) or ~5 × 10^−14^ m^2^/s (O15) at 0.1 gH_2_O/gdw and below. An EPR and ST-EPR spectroscopic investigation by Buitink *et al*.^7^ has suggested that in pollen the mobility of a polar nitroxide spin probe decreased as the water content decreased from 0.2 gH_2_O/gdw (the highest water content investigated in their study) to 0.1 gH_2_O/gdw at any temperatures under investigation. The mobility reached a minimum between 0.05 and 0.1 gH_2_O/gdw, but increased again when the water content was further decreased below 0.05 gH_2_O/gdw. The same study showed that in pea axes the mobility slightly increased again or reached a constant level at water content below 0.05 gH_2_O/gdw. The experimental observations for pea axes and pollen are similar to our findings for the trehalose-water matrix, even though it is evident that the diffusion coefficient profile in the water-deficit region is dependent on the positions of atoms under evaluation. For example, the diffusion coefficients of O16 and HO16 are noticeably higher than those of O11, O13 and O15. This is mainly because that the O16 and HO16 atoms are the most distant from the ring backbone and thus may have more flexibility. In contrast, the O11 atom forms the glycosidic linkage between the two rings and therefore has the lowest translational mobility. The molecular mobility can be significantly decreased when water clusters are removed from the matrix as seen in the range of 0.5–2 gH_2_O/gdw. But the formation of interconnected bulky trehalose proposed earlier can account for the slight change in mobility within the range 0.02–0.1 gH_2_O/gdw. Given that the bound water could act as bridges between trehalose molecules and fill in the free volume in the trehalose bulk, the bound water can actually help constrain the flexibility of the trehalose structure. However, the mobility of the trehalose molecules can increase due to the loss of water bridges and the presence of free volumes when the bound water molecules are excessively depleted. As seen in Fig. 7(b), the probability of locating O atoms of water molecules within a ~2.75 Å radius of O16 atoms is higher than locating them around any of the other O atoms of trehalose molecules. In other words, when the water content is 0.1 gH_2_O/gdw and below, the remaining water molecules prefer to H-bond with O16 compared to other oxygen atoms in trehalose, as evidenced by the *g*(*r*) profiles in Fig. 7(b), which supports the explanation that the increase in diffusion coefficients of O16 and HO16 below 0.02 gH_2_O/gdw could be attributed to the loss of constraints provided by bound water.

In summary the simulation in this study predicts that during the dehydration of the trehalose-water matrix the trehalose molecules may form such a structure that trehalose molecules interconnect with each other as a networked structure with one or two bound water molecules scattered throughout the network. By the time the water content reaches 0.1 gH_2_O/gdw, this structure has developed substantially. When the amorphous, fully or partially dehydrated matrix is exposed to a certain relative humidity, water molecules will percolate the bulky trehalose matrix. In the beginning, absorbed water molecules will form H-bonds with trehalose, without generating free water. Beyond a critical point, such as 0.1 gH_2_O/gdw suggested by this study, some of the absorbed water molecules become free by isolating themselves from trehalose molecules in the form of water clusters that have high molecular mobility, thereby increasing the probability of deteriorating the bulky matrix. Therefore, for applications requiring optimal suppression of molecular mobility it is suggested that for a binary trehalose-water matrix the water content should be maintained at ~0.1 gH_2_O/gdw. In the context of preservation, the water bound to trehalose may be necessary to constrain the motions of embedded active agents, such as proteins. We showed that the diffusion coefficient of oxygen atoms in the trehalose molecules decreased during dehydration as long as the water content was maintained above a certain level, however the diffusion coefficient of some of the oxygen atoms increased again with further removal of the bound water molecules. The removal of the bound water from the trehalose network could actually adversely affect preservation outcome by removing localized ‘molecular restraints’. For example, an increase in molecular mobility at very low moisture contents could allow side chain flexibility in embedded proteins that could lead to loss of function.

In the case of larger constructs, such as cells, the rapid increase in size of the trehalose network together with a reduction in overall volume as the water content is decreased below 0.5 gH_2_O/gdw could lead to the compression or shearing of cells as the matrix rigidifies. In the current study, membrane integrity is lost within the same narrow range of moisture contents within which the matrix becomes highly networked and loses molecular flexibility. Cells are also observed to be sequestered within channels as the moisture content progresses to this level. Further studies to examine the effect of additives on the networking characteristics of trehalose may yield optimized preservation formulations for cells and other complex constructs.

The general goal for dehydration processing is to attain a moisture content that is low enough to ensure that molecular mobility is significantly diminished at the desired storage temperature, while minimizing the loss of viability due to chemical and physical stresses that accumulate during processing. Using the Gordon-Taylor equation as a guide (Fig. 3), this would suggest that samples will need to be dried to a moisture content of 0.14 gH_2_O/gdw or below to achieve a *T*_*g*_ (and thus storage temperature) above 0 °C. Although this is a general rule of thumb, seed storage studies have provided evidence for a ‘crossover temperature’ that is higher than the *T*_*g*_, yet is consistent with extended shelf life^24^. It is possible that higher moisture contents could still yield a suitably stable non-refrigerated product, and this would need to be evaluated in future studies.

In the current study we examined direct and immediate injury during processing in order to understand its relationship to the molecular organization within the trehalose glass as this state develops, and to identify critical processing limits. Our studies suggest that when dehydrating cells in a trehalose-based composition the cell viability is significantly diminished below a moisture content of 0.30 gH_2_O/gdw and approaches zero viability as the moisture content reaches 0.10 gH_2_O/gdw, consistent with compression and physical stress as trehalose forms a large interconnected network. Further studies would be necessary to elucidate chemical processing injury that might not be detected with membrane integrity assays, as these studies might indicate a moisture content limit that is higher than observed in the current studies. Although further studies are needed to determine the shelf life of cells at different moisture contents and storage conditions, the current studies indicate a lower limit of moisture content that can be tolerated during processing and the physical basis for this processing limit.

## Conclusions

Dry preservation of biologics is suggested to be a cost-effective alternative to cryopreservation. Identifying the optimal water content for preservation outcomes is an essential component of successful implementation of dry preservation methods. By conducting MD simulations, we investigated the hydrogen-bonding characteristics and clustering phenomenon in trehalose-water mixtures in the water content range of 0–2 gH_2_O/gdw. The membrane integrity assay showed that the post-rehydration viability decreased dramatically within the range of 0.1–1 gH_2_O/gdw for T cells that were dried in trehalose-based media. Our MD simulations showed that the H-bonding capacity of trehalose, denoted as 
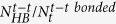
 correlated closely with the *T*_*g*_ profile as a function of moisture content. The aqueous trehalose compositions below 0.1 gH_2_O/gdw contained a giant cluster of over 200 trehalose molecules, but trehalose molecules in composition with water at values over 0.1 gH_2_O/gdw tend to form only isolated, small-sized clusters. It was suggested that as the water content approaches 0.1 gH_2_O/gdw the matrix forms an interconnected trehalose skeleton with only one or two bound water molecules scattered in the bulk, which is responsible for the substantially slowed-down increase of glass transition temperature in this region. As this interconnected network develops, cell viability also diminishes very significantly, and cells are observed to accumulate in channels, suggesting that cells are being compressed into increasingly smaller voids as the network rigidifies. For atoms O16 and HO16 that are the most distant from the glucose rings, their diffusion coefficients are found to fluctuate within the range of 0.02–0.1 gH_2_O/gdw and increase again at 0.01 gH_2_O/gdw and below, due to the loss of water filling in free volumes among trehalose molecules. The findings in this study provided detailed insights into the structural characteristics and dynamics of dried preservation compositions, which can benefit the optimal design of dry preservation protocols.

## Materials and Methods

### Cell culture

Jurkat Clone E6-1 cells (ATCC, Manassas, VA), which are acute leukemia T cells from a human male, were maintained in RPMI-1640 (ATCC, Manassas, VA) with 10% FBS (HyClone, Logan, UT) and 1% Penicillin (Mediatech Inc., Herndon, VA) in T-flasks (Sarstedt Inc., Newton, NC) at 37 °C with 5% CO_2_.

### Preparation of drying solutions

Two types of drying solutions were used to evaluate their effect on cell survival during drying: 1) 200 mM trehalose (high purity α, α-trehalose dihydrate from Ferro Pfanstiehl Laboratories Inc., Waukeg, IL) in 0.33X PBS (Gibco BRL Life Technologies Inc., Grand Island, NY), (310 mOsmol/kg and pH 7.20) and 2) 300 mM trehalose in deionized water (300 mOsmol/kg and pH 5.75). The osmolality was measured by a vapor-pressure osmometer (Wescor, Logan, UT) and the pH was measured by a SevenMulti pH meter (Mettler Toledo, Switzerland).

### Microwave-assisted drying process

For each experimental run cells were prepared at a concentration of 2.5–3 × 10^6^ cells/ml in the drying solution. An aliquot of 40 μl was pipetted onto a glass coverslip (Fisher Scientific, Pittsburg, PA) which was placed on a custom turntable made from ultra-high molecular weight polyethylene^12^. The turntable was loaded into a SAM 255 microwave (CEM Corp., Matthews, NC) for processing at a power setting of 19% (nominally 146 W). The microwave was accommodated within a custom-made environmental chamber which was maintained at a relative humidity of 11 ± 2.5%. In order to cover a relatively wide range of end water content, the 200 mM trehalose plus 0.33X PBS samples were dried in the microwave for 27–28 min, which yielded moisture contents in the range 0.1–4 gH_2_O/gdw. The 300 mM trehalose samples were processed for 27–35 min to achieve the same range.

### Determination of water content

To determine the water content of dried samples, the mass of each sample was measured before and after microwave-assisted drying using an AX105 DR Analytical balance (Mettler Toledo, Switzerland). The solid contents of each solution was determined by bake-out of known quantities of solution using a gravity convection oven (VWR, West Chester, PA) at 90 °C for 48 hours. Following this drying period, the samples were transferred to desiccation chambers containing phosphorus pentoxide, and held there until cooled to room temperature. Final mass was recorded and used to calculate the percent solids of each drying solution, based on the average of five replicates. All experimental measurements were expressed in terms of the mass of water in the sample per mass of solids in the starting solution (gH_2_O/gdw). Cells were assumed to contribute negligibly to the dry weight of solutions.

### Viability assessment

Immediately after the microwave processing and mass measurement each sample was placed onto a 35 mm culture dish (Sarstedt Inc., Newton, NC) and then rehydrated with 2 ml of full-complement media containing two stains, the cell-permeable nucleic acid stain Hoechst 33342 and the cell impermeable stain Sytox Green (Life Technologies, Carlsbad, CA). The samples were then placed in an incubator held at 37 °C with 5% CO_2_ for 30 min. The viability was determined by counting the numbers of total cells (stained with Hoeschst 33342) and dead cells (stained with Sytox Green) in 5 representative images, using an Olympus IX80 inverted microscope (Olympus America Inc., Melville, NY) with the associated filter cubes for each stain.

### MD simulation methods

A specified number of water molecules (0–8209) were randomly mixed with 216 trehalose molecules to generate simulation systems having water contents of 0, 0.01, 0.02, 0.05, 0.1, 0.5, 1, and 2 gH_2_O/gdw. Each simulation box was equilibrated for 20 ns at 483 K for the compositions of ≤ 0.1 gH_2_O/gdw and at 370 K for compositions of >0.1 gH_2_O/gdw to achieve complete solvation. Each simulation system was then quenched to 100 K at 500 K/ns before it was annealed to 150 K or 295 K at 5 K/ns. The annealed system was equilibrated at the end temperature for another 10 ns. At least three sets of frames were extracted from the last 5 ns trajectory for statistical analysis in the form of mean ± standard deviation. The all-atom CHARMM36 force fields for α,α-trehalose^25^ and the modified TIP3P water model^26^ were employed. All simulations were carried out using the NAMD simulation program^27^ with the isothermal-isobaric (NPT) ensemble at 101.325 kPa. The temperature was fixed using Langevin dynamics^28^ with a damping coefficient of 1 ps^−1^ and the pressure was fixed by a modified Nosé-Hoover method which is a combination of the constant pressure algorithm proposed by Martyna *et al*.^29^ with piston fluctuation control implemented using Langevin dynamics^30^. Periodic boundary conditions were applied. The time step was 2 fs. Other details of the MD simulations can be found in our previous studies^22^,^31^. Hydrogen bonds in the aqueous trehalose mixtures were identified by the geometric criteria. A certain aggregate between two oxygen atoms was regarded as an H-bond only if the distance between them did not exceed 3.5 Å, which is the position of the first minimum of the radial distribution function (*g*_*oo*_(*r*)), and the angle O-H···O was greater than 150°, based on the preferential linearity of H-bonds^23^,^32^.

## Additional Information

**How to cite this article**: Weng, L. *et al*. Effects of Water on Structure and Dynamics of Trehalose Glasses at Low Water Contents and its Relationship to Preservation Outcomes. *Sci. Rep.* **6**, 28795; doi: 10.1038/srep28795 (2016).

## Figures and Tables

**Table 1:** The values of fitting parameters (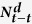 and *k*_2_) in Eq. (2) and goodness of fitting (*R*^2^).

*T*	*k*_*2*_	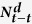	*R*^*2*^
150 K	3.4 ± 0.2	1.19 ± 0.05	0.9983
295 K	2.8 ± 0.2	1.12 ± 0.05	0.9987
